# The 2014 updated version of the Confusion Assessment Method for the Intensive Care Unit compared to the 5th version of the Diagnostic and Statistical Manual of Mental Disorders and other current methods used by intensivists

**DOI:** 10.1186/s13613-018-0377-7

**Published:** 2018-03-01

**Authors:** Gérald Chanques, E. Wesley Ely, Océane Garnier, Fanny Perrigault, Anaïs Eloi, Julie Carr, Christine M. Rowan, Albert Prades, Audrey de Jong, Sylvie Moritz-Gasser, Nicolas Molinari, Samir Jaber

**Affiliations:** 1 Department of Anaesthesia and Critical Care Medicine, University of Montpellier Saint Eloi Hospital, 80, avenue Augustin Fliche, 34295 Montpellier Cedex 5, France; 2 PhyMedExp, INSERM U1046, CNRS, UMR 9214, University of Montpellier, Montpellier, France; 3 Department of Medicine, Division of Allergy, Pulmonary, and Critical Care Medicine and the Center for Health Services Research, Vanderbilt University School of Medicine, Nashville, TN USA; 4 Geriatric Research Education Clinical Center (GRECC), Department of Veterans Affairs, Tennessee Valley Healthcare System, Nashville, TN USA; 5 Department of Speech and Language Therapy, School of Medicine, University of Montpellier, Montpellier, France; 6 Institute of Neurosciences of Montpellier, INSERM U105, University of Montpellier, Montpellier, France; 7 Department of Statistics, University of Montpellier Hospitals, Montpellier, France

**Keywords:** Delirium, Intensive care unit, Critical care

## Abstract

**Background:**

One third of patients admitted to an intensive care unit (ICU) will develop delirium. However, delirium is under-recognized by bedside clinicians without the use of delirium screening tools, such as the Intensive Care Delirium Screening Checklist (ICDSC) or the Confusion Assessment Method for the ICU (CAM-ICU). The CAM-ICU was updated in 2014 to improve its use by clinicians throughout the world. It has never been validated compared to the new reference standard, the Diagnostic and Statistical Manual of Mental Disorders 5th version (DSM-5).

**Methods:**

We made a prospective psychometric study in a 16-bed medical–surgical ICU of a French academic hospital, to measure the diagnostic performance of the 2014 updated CAM-ICU compared to the DSM-5 as the reference standard. We included consecutive adult patients with a Richmond Agitation Sedation Scale (RASS) ≥ −3, without preexisting cognitive disorders, psychosis or cerebral injury. Delirium was independently assessed by neuropsychological experts using an operationalized approach to DSM-5, by investigators using the CAM-ICU and the ICDSC, by bedside clinicians and by ICU patients. The sensitivity, specificity, positive and negative predictive values were calculated considering neuropsychologist DSM-5 assessments as the reference standard (primary endpoint). CAM-ICU inter-observer agreement, as well as that between delirium diagnosis methods and the reference standard, was summarized using *κ* coefficients, which were subsequently compared using the *Z*-test.

**Results:**

Delirium was diagnosed by experts in 38% of the 108 patients included for analysis. The CAM-ICU had a sensitivity of 83%, a specificity of 100%, a positive predictive value of 100% and a negative predictive value of 91%. Compared to the reference standard, the CAM-ICU had a significantly (*p* < 0.05) higher agreement (*κ* = 0.86 ± 0.05) than the physicians,’ residents’ and nurses’ diagnoses (*κ* = 0.65 ± 0.09; 0.63 ± 0.09; 0.61 ± 0.09, respectively), as well as the patient’s own impression of feeling delirious (*κ* = 0.02 ± 0.11). Differences between the ICDSC (*κ* = 0.69 ± 0.07) and CAM-ICU were not significant (*p* = 0.054). The CAM-ICU demonstrated a high reliability for inter-observer agreement (*κ* = 0.87 ± 0.06).

**Conclusions:**

The 2014 updated version of the CAM-ICU is valid according to DSM-5 criteria and reliable regarding inter-observer agreement in a research setting. Delirium remains under-recognized by bedside clinicians.

**Electronic supplementary material:**

The online version of this article (10.1186/s13613-018-0377-7) contains supplementary material, which is available to authorized users.

## Background

Nearly one third of patients admitted to an intensive care unit (ICU) will develop delirium [1], which is subsequently associated with sedation–analgesia management issues [2–4], an increased duration of mechanical ventilation, length of stay in the ICU and hospital, risk of death, as well as of having long-term neurocognitive dysfunction [1, 5]. Guidelines recommend the routine use of validated clinical tools for the early recognition and treatment of delirium by medical and nursing ICU teams, even if they are not expert neuropsychologists [6].

Among the delirium diagnosis tools that can be used by ICU clinicians in routine practice, the Confusion Assessment Method for the ICU (CAM-ICU) [7, 8] and the Intensive Care Delirium Screening Checklist (ICDSC) [9] have been extensively studied for more than 15 years, demonstrating good psychometric properties in a research setting [6]. In 2014, the CAM-ICU and its training manual were updated to avoid any misinterpretation by users (Table 1). Also, the original version of the CAM-ICU [7, 8] was validated against the American Psychiatric Association’s fourth edition of the Diagnostic and Statistical Manual of Mental Disorders (DSM-IV). Differences between the 4th and the new 5th versions (DSM-5) regarding delirium assessment are still under debate [10, 11].

**Table 1:** Principal changes made in the 2014 updated version of the CAM-ICU training manual

Features	Changes in the 2014 updated version
Feature 1 = *Acute onset or fluctuating course of mental status*	The term “sedation level” was intertwined with the “level of consciousness” throughout the method because some clinicians used these two terms interchangeably, but others were confused by the fact that patients could not receive sedatives. Note that RASS can be used in patients sedated or non-sedated
Feature 2 = *Inattention*	Another new 10-letter set (C–A–S–A–B–L–A–N–C–A) is now provided to allow for international understanding
Feature 3 = *Altered level of consciousness*	Following many institutions, the former feature #3 (disorganized thinking) was switched with former feature #4 (altered level of consciousness). The new feature #3 (level of consciousness) is often sufficient to rate a CAM-ICU as positive, while the new feature #4 (disorganized thinking) is less often necessary to perform in the end
Feature 4 = *Disorganized thinking*	This feature was rewritten to avoid any confusion in the total number of errors required among the 4 questions and 1 command: > 1 error = feature #4 present
Supporting materials	The updated method was associated with a 32-page complete training manual (available at www.icudelirium.org), including an extensive Frequently Asked Questions section, new case studies and links to the ICUDelirium.org Web site that was completely remodeled

*CAM-ICU* Confusion Assessment Method for the Intensive Care Unit, *RASS* Richmond Agitation Sedation Scale

The primary objective of this study was to measure the ability of the 2014 updated version of the CAM-ICU to diagnose delirium according to the most updated neuropsychological reference standard, i.e., the DSM-5 method. Secondary objectives were (1) to measure inter-observer agreement for the CAM-ICU, and (2) within the context of a comprehensive investigation of delirium assessment in a real-life intensive care setting, to compare the diagnostic accuracy of the CAM-ICU to the ICDSC and to physician, resident and nurse recognition of delirium, as well as to common orientation questions and to the patient’s own impression of feeling delirious.

## Methods

### Ethics and consent

The protocol was approved by an independent ethics committee [*Comité de Protection des Personnes (CPP) Sud Méditerranée.IV* (N°ID-RCB: 2015-A01084-45; Protocol version 1: 06/23/2015)] and was conducted in accordance with the Declaration of Helsinki (clinicaltrials.gov: NCT02760446). Written consent was required from the patient or the legally authorized representative or a proxy/surrogate decision maker (patient’s next of kin) who gave consent on the patient’s behalf, followed by the patient’s consent as soon they could communicate.

### Population

The study took place in the 16-bed medical–surgical ICU of the University of Montpellier Saint Eloi Hospital, an academic tertiary-care hospital, from November 2015 to April 2016. All consecutive French-speaking patients ≥ 18-year old were eligible for enrollment if they had a Richmond Agitation Sedation Scale (RASS) ≥ −3 [12–14]. Exclusion criteria were preexisting cognitive disorder/psychosis (baseline cognitive status is often unknown early in the ICU stay, precluding accurate evaluation of change in mental status, a key feature of delirium), visual/hearing loss without helpers, pregnancy (according to French law), patients under tutelage, withdrawal of consent or change in clinical status that would preclude a complete cognitive testing.

### Study conduct

**Fig. 1:**
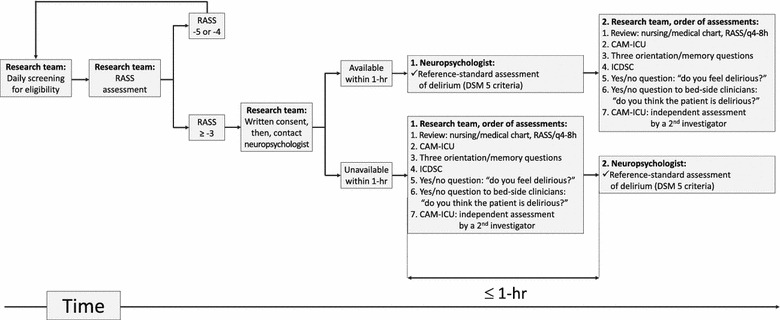
Study design. The order of assessments by the research team was determined to check both the patient’s eligibility and the presence of some CAM-ICU and ICDSC features (i.e., fluctuating course of mental status assessed by RASS ratings). ICDSC was assessed after CAM-ICU because ICDSC included some CAM-ICU features (i.e., inattention). *RASS* Richmond Agitation Sedation Scale, *CAM-ICU* Confusion Assessment Method for the Intensive Care Unit, *ICDSC* Intensive Care Delirium Screening Checklist, *DSM-5* 5th version of the Diagnostic and Statistical Manual of Mental Disorders

All consecutive patients admitted to our ICU were screened by the ICU research team every morning including weekends, until they reached the inclusion criteria during a period of 5 months (November 2015–March 2016). After having obtained consent and enrolling the patient, the ICU research team contacted one of two neuropsychological experts participating in the study to independently perform a neuropsychological assessment of delirium. Figure 1 summarizes the timing of delirium assessments by the neuropsychological experts and the ICU research team.

### Data collection

#### Delirium

Delirium was assessed once, the same day, in five ways that occurred as close together as possible in time, but strictly independent of each other. Separate clinical research forms were used to assure independence between observers.

##### 1. ICU delirium tools: CAM-ICU and ICDSC

The ICU research team used the French versions of the 2014 updated CAM-ICU training manual and the ICDSC [9, 15]. The CAM-ICU was assessed by two independent investigators to estimate inter-observer agreement.

##### 2. Expert neuropsychological assessment of delirium (the reference standard)

 The neuropsychological experts were members of the speech and language therapy team, usually in charge of neuropsychological testing in the neurology/neurosurgery/neuro-ICU departments of the Neurosciences University Hospital of Montpellier. A standardized method for diagnosing delirium was used based on the DSM-5 [16] using the Montreal Cognitive Assessment (MOCA) [17], Dubois’ 5-word test [18], Language Screening Test (LAST) [19], with helpers for intubated ICU patients (see Additional file 1: Supplemental Digital Content).

##### 3. Bedside–clinician assessment of delirium

When immediately available, the patient’s bedside ICU team (i.e., the patient’s nurse, resident and attending physician) were contacted by the ICU research team to get their personal feeling about the presence or absence of delirium.

##### 4. The 3 simple orientation/memory questions for the assessment of delirium

The ICU research team also assessed delirium by asking three simple questions commonly used to assess delirium at our institution: Where are you? What day is it today? Who is the president? (because long-term memory is frequently altered in delirium) [20]. The number of incorrect and absent response(s) was recorded.

##### 5. The patient’s own feeling

At the end of testing, the patients were asked by the ICU research team if they had the impression they were confused.

#### Demographic and medical data

Age, gender, comorbidities and the reason for ICU admission were recorded. The Simplified Acute Physiological Score II (SAPS-II) score [21] and the Sequential Organ Failure Assessment (SOFA) score [22] were calculated within 24 h after ICU admission and upon enrollment. In case enrollment occurred before 24 h, the SAPS-II score took into account the worst value available during the 24 h preceding enrollment. Therapeutics such as sedation, mechanical ventilation and the use of vasopressors were collected upon enrollment.

### Data presentation and statistical analysis

#### Psychometric properties of the CAM-ICU

##### Validity (primary endpoint)

The performance of the CAM-ICU for diagnosing delirium was assessed by measuring the sensitivity, specificity, positive predictive value (PPV) and negative predictive value (NPV) according to standardized definitions [23, 24]. Expert assessments were used as the reference standard.

##### Reliability

The kappa coefficient was calculated between the two ICU research investigators. Kappa coefficients above 0.80, 0.60 and 0.40 are considered as measuring ‘near perfect,’ ‘strong’ and ‘moderate’ levels of agreement [25], respectively.

#### The diagnostic performance of other methods commonly used to assess delirium (ICSDC, bedside clinician assessments, 3-question test), as well as patients’ impressions)

The sensitivity, specificity, PPV and NPV were also calculated using the expert assessments as the reference standard. To compare all five methods for diagnosing delirium, kappa coefficients were calculated between the expert assessments and the other methods. Kappa coefficient comparisons between methods were made using the *Z*-test [26]. A *p* value of < 0.05 was considered statistically significant.

##### Power analysis

The study sample size was determined in relation to the primary endpoint. For expected values [8] of sensitivity and specificity at 85 and 75%, respectively, a desired level of precision set at 10% and the prevalence of delirium set at 50%, the number of patient inclusions required for achieving appropriate power would be 95. Taking into account possible post-enrollment exclusions, 115 patients needed to be enrolled in the study. The prevalence of delirium ranged from 30 to 90% in the literature [1]. Thus, we set the prevalence of delirium at 50% which is conservative regarding the number of patients needed to be analyzed, in order to maximize the power.

##### Data presentation

Quantitative data are shown as medians and 25th–75th percentiles. Data were analyzed using SAS version 9.2 (SAS Institute, Cary, NC).

## Results

A total of 108 patients were included for analysis among the 115 patients enrolled in the study. A Standards for Reporting of Diagnostic Accuracy (STARD) diagram for patient enrollment is shown in Additional file 1: Supplemental Digital Content. Table 2 summarizes patient demographic and medical characteristics. Delirium was diagnosed by the neuropsychological experts for 41 of the 108 patients (38%).

**Table 2:** Demographic and medical characteristics of the 108 patients included for analysis

Characteristics	Median [IQR] or *n* (%)
Upon ICU admission	
Age (years)	62 [54–68]
Sex [*n* (%)]	
Male	64 (59%)
Female	44 (41%)
Body mass index (kg/m^−2^)	25 [23–29]
Type of admission	
Unplanned surgical (from operating room) [*n* (%)]	26 (24%)
Planned surgical (from operating room) [*n* (%)]	15 (14%)
Surgical (from ward) [*n* (%)]	5 (05%)
Medical [*n* (%)]	62 (57%)
SAPS II score	39 [31–49]
SOFA score	7 [4–9]
Sepsis at admission [*n* (%)]	47 (44%)
Intubation at admission [*n* (%)]	70 (65%)
Upon study enrollment	
Time between ICU admission and enrollment (days)	3 [2–5]
SAPS II score	29 [23–38]
SOFA score	4 [2–7]
Vigilance status	
Median RASS level	0 [0–0]
RASS level = +2 [*n* (%)]	2 (2%)
RASS level = +1 [*n* (%)]	8 (7%)
RASS level = 0 [*n* (%)]	76 (70%)
RASS level = −1 [*n* (%)]	13 (12%)
RASS level = −2 [*n* (%)]	4 (4%)
RASS level = −3 [*n* (%)]	5 (5%)
Therapeutics	
Invasive mechanical ventilation [*n* (%)]	23 (21%)
Noninvasive mechanical ventilation [*n* (%)]	8 (07%)
Vasopressors [*n* (%)]	18 (17%)
Sedation (propofol) [*n* (%)]	12 (11%)
At ICU discharge	
Total duration of mechanical ventilation (days)	1.6 [0.3–5.5]
ICU length of stay (days)	6.5 [3.0–12.4]

*BMI* body mass index, *ICU* Intensive Care Unit, *IQR* inter-quartile range, *RASS* Richmond Agitation Sedation Scale, *SAPS II* Simplified Acute Physiological Score II, *SOFA* Sequential Organ Failure Assessment score

### Validation of the 2014 updated CAM-ICU

A positive CAM-ICU was found for 34 (31%) patients. Compared to expert assessments, there were 7 misclassified CAM-ICU ratings among the 108 ratings, of which there were 7 false negatives and no false positives. Compared to expert assessments, the CAM-ICU had a sensitivity of 83% [95% confidence interval 71–94], a specificity of 100% [100–100], a PPV of 100% [100–100] and a NPV of 91% [84–97].

To measure the inter-observer reliability of the CAM-ICU, 98 patients were assessed by a second investigator. For the ten remaining patients, a second assessment was impossible because of changes in vigilance status or clinical condition. The kappa coefficient for inter-observer reliability was 0.87 (SD ± 0.06) demonstrating strong agreement. There were no significant differences between the 5 ICU investigators and the 2 neuropsychological experts (kappa coefficients ranging from 0.82 ± 0.1 to 0.88 ± 0.2). First and second CAM-ICU investigators obtained similar agreement with experts’ assessments (kappa coefficients = 0.86 ± 0.1 and 0.85 ± 0.1, respectively).

### Diagnostic performance of commonly used methods for assessing delirium

Table 3 presents the statistical measurement of performance regarding delirium recognition via the CAM-ICU, and the ICDSC, by nurses, residents and physicians, as well as via three simple questions. The CAM-ICU demonstrated good performance, while the 3 simple questions demonstrated poor performance. The 3 simple questions demonstrated the highest sensitivity for a false or absent response, but with the lowest specificity and PPV. The ICDSC and clinicians’ diagnosis demonstrated similar performances.

**Table 3:** Patients’ clinical diagnosis and simple orientation questions

Measurement	ICU delirium tools		Patients’ clinician diagnosis^a^			3 Simple orientation questions			
	CAM-ICU	ICDSC	Physician	Resident	Nurse	1 false or absent response	2 false or absent responses	1 false response^b^	2 false responses^b^
Sensitivity	83% [71–94]	83% [71–94]	79% [65–94]	68% [52–83]	70% [54–86]	90% [81–99]	68% [54–83]	78% [59–97]	28% [7–48]
Specificity	100% [100–100]	87% [78–95]	85% [75–96]	93% [86–100]	89% [82–97]	66% [54–77]	82% [73–91]	73% [62–85]	92% [85–99]
PPV	100% [100–100]	79% [67–91]	79% [65–94]	85% [72–99]	78% [62–93]	62% [49–74]	70% [56–84]	47% [29–65]	50% [19–81]
NPV	91% [84–97]	89% [82–97]	85% [75–96]	82% [72–92]	85% [76–94]	92% [84–99]	81% [72–90]	92% [84–99]	81% [72–90]
Delirium diagnosis by non experts	31%	40%	41%	31%	31%	56%	37%	38%	13%
by experts	38%	38%	41%	39%	34%	38%	38%	21%	21%
Agreement with experts (*κ* ± SD)^c^	0.86 ± 0.05	0.69 ± 0.07	0.65 ± 0.09	0.63 ± 0.09	0.61 ± 0.09	0.51 ± 0.08	0.51 ± 0.09	0.41 ± 0.10	0.23 ± 0.13

The statistical measurement of performance is expressed as the percentage and its 95% confidence interval. *CAM-ICU* Confusion Assessment Method for the Intensive Care Unit, *ICDSC* Intensive Care Delirium Screening Checklist, *PPV* positive predictive value, *NPV* negative predictive value

^a^Among the 108 patients included for analysis, patients’ clinicians were available upon study completion for 91 patient nurses (84%), 89 patient residents (82%) and 71 patient physicians (66%). Among the clinicians participating in the study, the 4 nurses, 1 resident and 1 physician who could not answer the question whether the patient was or was not delirious were not taken into account for the analysis

^b^Analysis was performed among the 78 patients (72%) who were able to answer all three simple questions

^c^The agreement between each method of delirium diagnosis and the assessment by the neuropsychological experts using DSM-5 criteria (reference standard) was measured using kappa coefficients

**Fig. 2:**
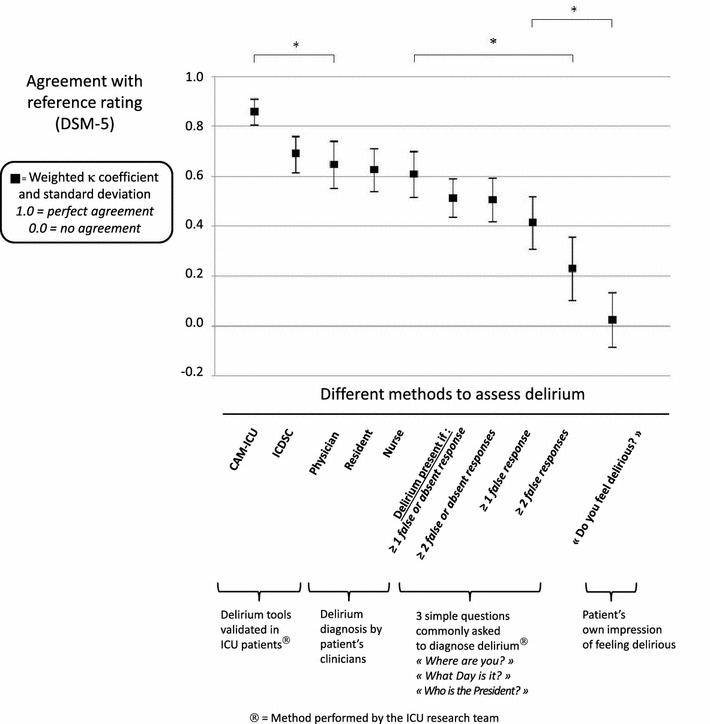
Agreement between different delirium assessment methods and the neurological experts’ reference rating using the DSM-5 criteria. This figure shows the graphic representation of kappa coefficients and their standard deviations for each of the methods used to assess delirium. The kappa coefficient measured the agreement between each of the methods and the assessment by the neuropsychologist experts using DSM-5 criteria (reference standard). For simple questions, we did not decide a priori how to analyze the answers. Because some patients answered some questions but did not answer other ones, we decided a posteriori to analyze these data following two approaches: including all patients and including only the patients able to answer all the questions. Several thresholds were tested, i.e., delirium was defined in all patients if they gave at least 1 or 2 false or no response(s), or, among the patients who were able to answer all simple questions, if the patients gave at least 1 or 2 false response(s). There was a significant difference (*p* < 0.047) between the CAM-ICU and each of the other methods, except the ICDSC (*p* = 0.054). There were significant differences between “all methods from CAM-ICU to ≥ 1 false response to simple questions” and “patient’s own impression of feeling delirious,” as well as between “all methods from CAM-ICU to nurse diagnosis” and “≥ 2 false responses to simple questions” or “patient’s own impression of feeling delirious.” *: Significant difference (*p* < 0.05); *CAM-ICU* Confusion Assessment Method for the Intensive Care Unit, *ICDSC* Intensive Care Delirium Screening Checklist

Figure 2 shows the graphic representation of kappa coefficients for each of the methods used to assess delirium. The kappa coefficient measured the agreement between each of the methods and the assessment by the neuropsychological experts using DSM-5 criteria (reference standard). There was a significant difference between the level of agreement found for the CAM-ICU (kappa 0.86 ± 0.05) and that found for other methods (*p* < 0.047), except the ICDSC (0.69 ± 0.07, *p* = 0.054, *Z*-test). Detailed data regarding CAM-ICU/ICDSC procedures are provided in Additional file 1: Supplemental Digital Content.

The 3 simple questions and the patient’s own impression had the lowest agreements with experts and demonstrated significant differences with other methods.

### Patient’s own impression of feeling delirious

Among the 108 patients, 77 (71%) were able to answer the question as to whether they felt delirious or not (all had a RASS level ≥ −1). Among these patients, 27 (35%) answered that they were. The patient’s sensitivity for recognizing delirium compared to expert assessments was 38%, with a specificity of 66%, a PPV of 22% and a NPV of 80%.

## Discussion

The main finding of this study is that the 2014 updated version of the CAM-ICU was valid compared to the DSM-5 reference standard, with strong inter-observer agreement. CAM-ICU and ICDSC agreed with experts’ opinion without significant difference. The CAM-ICU had superior performance for diagnosing delirium compared to the bedside–clinicians’ opinion, as well as compared to simple questions that are commonly used to assess delirium. Patient impressions of feeling delirious are not accurate, with a false-positive rate at 78%.

Delirium is multifactorial and frequent in critically ill ICU patients [6, 27–35]. It is diagnosed in 10–90% of ICU patients, depending on the diagnosis tool, the timing of assessment (during or after interrupting sedation), as well as the frequency of assessment (one-point assessment for validation studies or throughout the ICU stay) [6, 36]. A recent review of 42 studies estimated the prevalence of delirium at 5280 (31.8%) out of 16,595 critically ill patients [1]. The prevalence of delirium in the present study is close to this result: 38% according to neuropsychological assessment and 31% according to the CAM-ICU. Although frequent, delirium is under-recognized by both physicians and nurses [37]. Compared to the CAM-ICU, clinician sensitivity for diagnosing delirium is about 30% [38, 39]. The recognition of delirium by physicians and nurses in the present study was better, with a sensitivity of nearly 70%, suggesting that there might have been an increase in clinician awareness regarding delirium in the ICU over the past decade [40]. However, with PPVs under 80% and NPVs under 90% in the present study, clinicians should still use an ICU delirium tool to improve their diagnostic performance, according to current guidelines [6]. In the present study, agreement with expert diagnosis was not significantly different between the CAM-ICU and the ICDSC. In previous studies, the pooled sensitivity and specificity were 76 and 96%, respectively, for the CAM-ICU, and 80 and 75% for the ICDSC [41]. Sensitivity and specificity were slightly higher for both tools in the present study. This could be due to the study setting where a research team with experience in conducting research in the area of sedation–analgesia was available to conduct this psychometric study. Indeed, performance measurements for ICU delirium tools are higher in research settings than in real life [41]. However, the original study validating the CAM-ICU reported higher sensitivity and specificity than in the present study, with a sensitivity ranging from 93 to 100% and a specificity ranging from 98 to 100% [8]. This could be due to differences in the studied populations. In the original study [8], patients had a median Glasgow score of 7 at enrollment, while in the present study, 80% of patients had a RASS level ≥ 0, suggesting they were more alert. It has been reported that the CAM-ICU could have a lower sensitivity in alert patients, possibly because of better cognitive function [42, 43]. Because delirium prevalence and recognition may depend on the level of consciousness, some authors recommend stratifying delirium assessments for sedation score using a cutoff of RASS − 2 [44]. In the present study also, when taking into account only the 99 patients who had a RASS level of > −2 for a sensitivity analysis, the delirium prevalence was lower than in the overall population (32% instead of 38% according to the experts, 25% instead of 31%, according to the CAM-ICU). The CAM-ICU had a slightly lower sensitivity (78%, instead of 83%), while conserving the same specificity, positive and negative predictive values. Similar findings were obtained when excluding from the analysis the 12 patients who were sedated (8 of them having a RASS level of > −2).

Our study has several limitations. For example, all the methods for diagnosing delirium were assessed within a short time. This may have tired patients and decreased their cognitive functions. The research team planned the assessments within the space of an hour to increase the chance of measuring delirium at the same time for a given patient (Fig. 1). The agreement between the neuropsychological expert and the CAM-ICU was not significantly different whether the expert performed the assessment before or after the research team (kappa coefficient 0.86 ± 0.1 vs 0.85 ± 0.1, respectively). Secondly, except ICDSC, many other validated delirium tools [42] were not performed in order to make the duration of assessment feasible. In the same way, the ICDSC could have demonstrated higher sensitivity and specificity if it had been performed in more alert patients, and by the patient’s clinicians rather than by the research team. To perform the ICDSC, the research team took into account all nursing/medical charts (Fig. 1) but performed only “punctual” cognitive evaluations instead of evaluations over a nursing shift. The ICDSC was not performed by the patient’s clinicians to avoid any bias regarding their raw opinion about the presence or the absence of delirium. In other words, clinicians did not use a validated delirium tool which is to take into account for the interpretation of the data. The primary goal of the present study was to validate the 2014 updated version of the CAM-ICU. Measuring the psychometric properties of the ICDSC was only informative because it is a second recommended tool for assessing delirium and therefore frequently used throughout the world [6]. Moreover, the present study demonstrated no significant difference between the ICDSC and CAM-ICU regarding the agreement between the ICU research team and the neuropsychological experts (Fig. 2). However, this study was not calibrated to measure this difference. A longer period of evaluation could have resulted in a higher sensitivity for ICDS. Similarly, repeated measurements of delirium on a longer period of time could have lead to a higher sensitivity. Regarding the expert’s assessment, DSM-5 interpretations and use as a reference standard for delirium are a source of debate and thus may vary according to assessor [10, 11]. Finally, all the causes of delirium were not investigated because this was out of the scope of this psychometric study. Sepsis was present in 44% of patients at admission, and 11% of patients received sedatives at enrollment. Thus, a few intubated patients were included, due to a strategy of “early-sedation-interruption.” This study should be further performed in different settings/ICU populations.

Study strengths include the reference standard method used by experts to diagnose delirium, which was provided for the first time in detail to facilitate study reproducibility (see Additional file 1: Supplemental Digital Content). Aside from the expert assessment, a pragmatic approach for diagnosing delirium was also evaluated in order to reflect real-life situations in intensive care. This included nurse, resident and physician diagnoses, as well as commonly used simple orientation/memory questions. These questions are not appropriate for diagnosing delirium. This might be due to patient disorientation, which can be related to environment (absence of windows). The recommendation to use a validated delirium tool is reinforced by the fact that the ICU team is used to conducting clinical research and quality improvement projects in the area of agitation, sedation and analgesia [45–47]. Even in such an “a priori” favorable setting for the early recognition of delirium, bedside–clinicians still need to use a validated tool during their routine practice, repeatedly during the day and throughout the ICU stay. This is paramount for treating the factors associated with delirium [6, 27–35] as soon as possible, especially when taking into account the negative outcomes associated with delirium [5, 6]. A comprehensive approach [48] integrating delirium management with analgesia, sedation, mechanical ventilation, mobility/exercise and family engagement/empowerment has shown a positive impact on increasing ventilatory-free days [49, 50], decreasing delirium incidence [49–51] and improving hospital mortality [51].

 Finally, the study investigated the patient’s ability to recognize delirium. Though delusional memories are frequent in ICU survivors, they have not been investigated during hospitalization in the ICU setting [52–54]. Recent studies found no significant association between delirium in the ICU and mental disorders in survivors [55–57]. However, memories of being delirious in the ICU are associated with anxiety [56]. The link between delirium recollection, feelings of being delirious while in the ICU (which is possibly theoretically wrong or too abstract for some patients) and long-term psychological outcomes thus requires further exploration.

## Conclusions

 The 2014 updated version of the CAM-ICU is a valid tool for delirium diagnosis in a research setting in critically ill patients according to the DSM-5 criteria used by neuropsychological experts. It demonstrated important inter-observer reliability, and better performance for diagnosing delirium in ICU patients than physicians, residents and nurses, despite increased awareness regarding delirium in the ICU for many years. Future studies should investigate the discrepancies between validated methods to diagnose delirium (DSM-5, CAM-ICU, ICDSC) and the ICU team. Moreover, the patient’s own ability to report delirium might be inaccurate. Ethics committees should pay attention to delirium assessment when checking for patient’s ability to consent to participate in ICU studies [58]. This suggests also that delusional memories reported by survivors should be investigated in regard to a valid assessment of delirium during the ICU stay. In the ICU, patients should be asked about feeling delirious, comforted if they are not, but be taken care of regarding what could make them feel so.

## Additional file

**Additional file 1: Figure S1.** Standards for Reporting of Diagnostic Accuracy (STARD) diagram. **Figure S2**. Procedure data regarding the Confusion Assessment Method for the Intensive Care Unit (CAM-ICU). **Table S1**. Procedure data regarding the Intensive Care Delirium Screening Checklist (ICDSC). Protocol. Standardized neuropsychological assessment of delirium, adapted for critically ill patients. Helpers for intubated patients, used by neuropsychological experts.

## Abbreviations

CAM-ICU: Confusion Assessment Method for the Intensive Care Unit
DSM-5: 5th version of the Diagnostic and Statistical Manual of Mental Disorders
ICDSC: Intensive Care Delirium Screening Checklist
ICU: Intensive care unit
NPV: negative predictive value
PPV: positive predictive value
RASS: Richmond Agitation Sedation Scale
SAPS II: Simplified Acute Physiological Score II score
SOFA: Sequential Organ Failure Assessment
