# Supplementary material for: The 2014 updated version of the Confusion Assessment Method for the Intensive Care Unit compared to the 5th version of the Diagnostic and Statistical Manual of Mental Disorders and other current methods used by intensivists
Source: Ann Intensive Care. 2018 Mar 1;8:33. doi: 10.1186/s13613-018-0377-7 (PMC5833335; doi:10.1186/s13613-018-0377-7)
Supplement: Supplementary file 1 — Additional file 1: Figure S1. Standards for Reporting of Diagnostic Accuracy (STARD) diagram. Figure S2. Procedure data regarding the Confusion Assessment Method for the Intensive Care Unit (CAM-ICU). Table S1. Procedure data regarding the Intensive Care Delirium Screening Checklist (ICDSC). Protocol. Standardized neuropsychological assessment of delirium, adapted for critically ill patients. Helpers for intubated patients, used by neuropsychological experts. [file 13613_2018_377_MOESM1_ESM.pdf]

Supplemental Digital Content

This supplement has been provided by the authors to give readers additional information about their work.

Supplement to: Chanques et al., The 2014 updated version of the Confusion Assessment Method For The Intensive Care Unit Training Manual compared to the 5th version of the Diagnostic and Statistical Manual of Mental Disorders and other current methods used by intensivists

ClinicalTrials.gov number, NCT02760446

Table of contents

Supplemental Digital Content

Figure S1. Standards for Reporting of Diagnostic Accuracy (STARD) diagram p. 3

Figure S2. Procedure data regarding the Confusion Assessment Method for the Intensive Care Unit (CAM-ICU) p. 4

Table S1. Procedure data regarding the Intensive Care Delirium Screening Checklist (ICDSC) p. 5

Standardized neuropsychological assessment of delirium, adapted for critically ill patients p. 6

Helpers for intubated patients, used by neuropsychological experts p. 9

Figure S1. Standards for Reporting of Diagnostic Accuracy (STARD) diagram

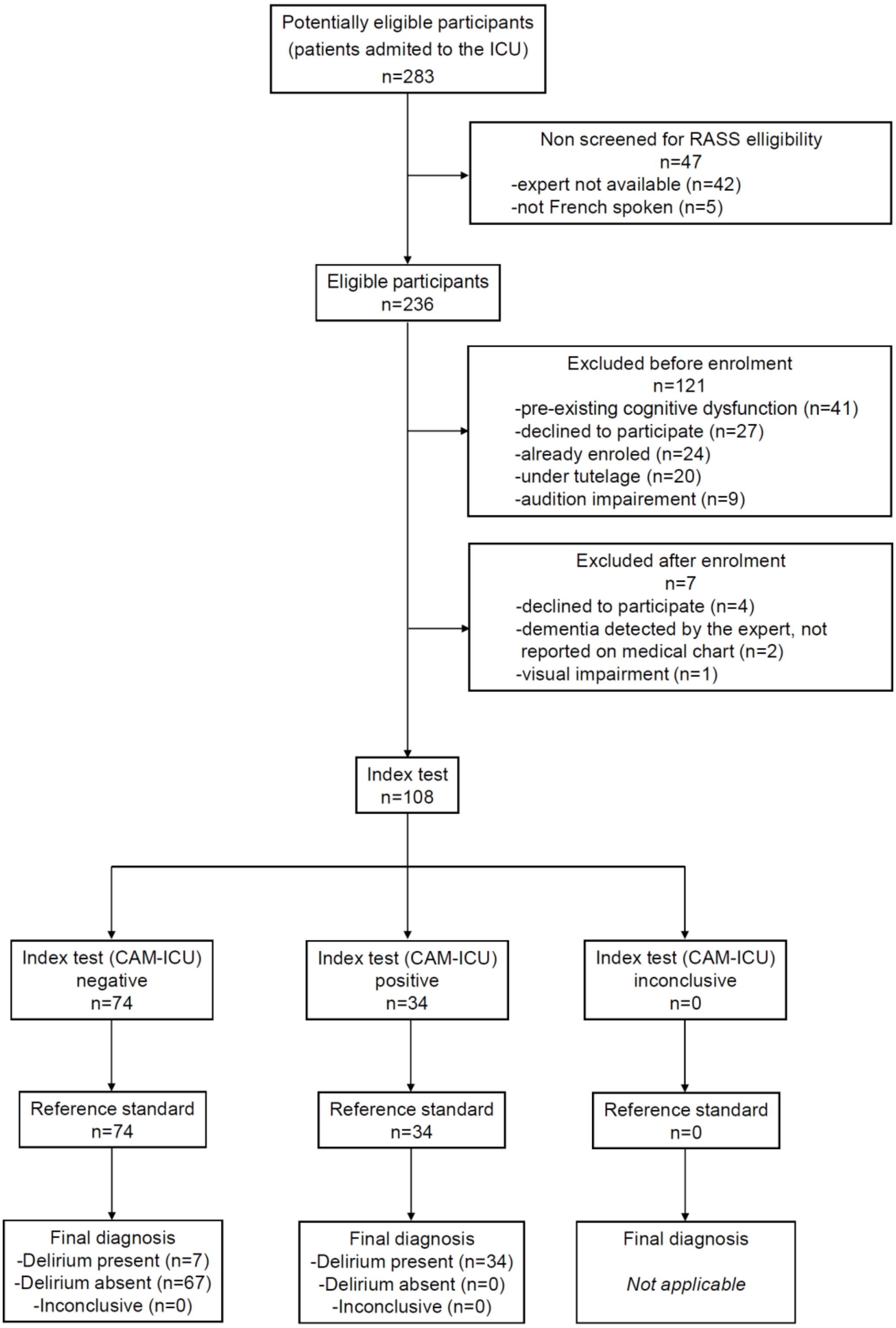

Screening and Assessment of Patients: 2015, November the 10th – 2016, March the 14th

RASS: Richmond Agitation Sedation Scale; CAM-ICU: Confusion Assessment Method for the Intensive Care Unit. Reference standard was the assessment by neuropsychological experts using the criteria of the 5th version of the Diagnostic and Statistical Manual of Mental Disorders.

Figure S2. Procedure data regarding the Confusion Assessment Method for the Intensive Care Unit (CAM-ICU)

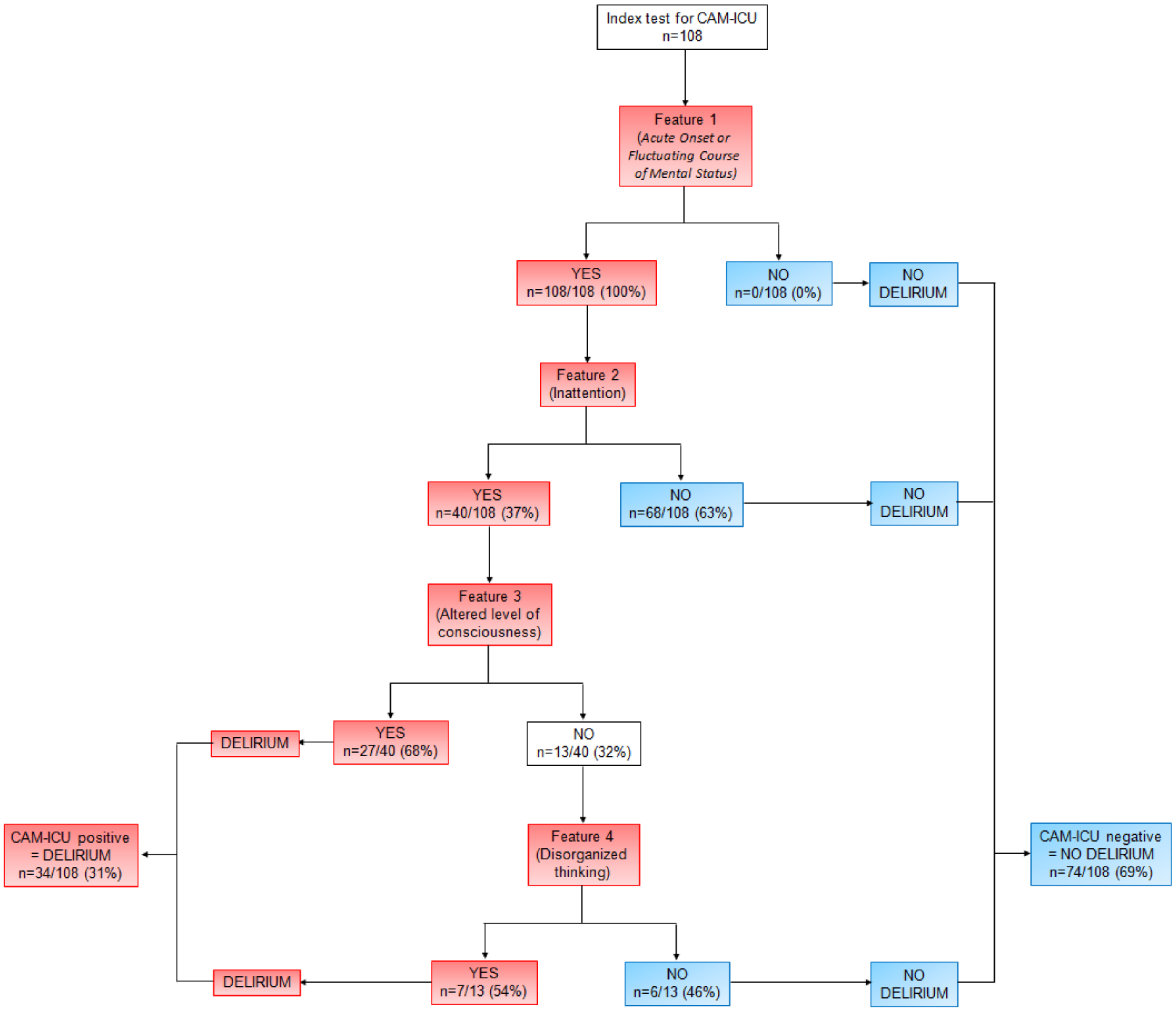

Table S1. Procedure data regarding the Intensive Care Delirium Screening Checklist (ICDSC)

**ICDSC items**	**Number of patients having an obvious manifestation of an item, among the 108 patients analysed, n (%)**
Altered level of consciousness	68 (63%)
Inattention	51 (47%)
Disorientation	40 (37%)
Hallucination, delusion or psychosis	2 (2%)
Psychomotor agitation or retardation	72 (67%)
Inappropriate speech or mood	13 (12%)
Sleep/wake cycle disturbance	19 (18%)
Symptom fluctuation	80 (74%)
**At least 4 items present among the 8 items = ICDSC positive = delirium**	**43 (40%)**

The ICDSC was performed by the research team at the end of a neuropsychological assessment including the Confusion Assessment Method for the Intensive Care Unit (CAM-ICU) and simple orientation/memory questions. Before this assessment: 1) the research team performed the Richmond Agitation Sedation Scale (RASS) to screen patients for eligibility; 2) presented the purpose of the study (including explanations about delirium) to eligible patients in order to obtain their signed consent to participate into this research (or to the surrogate decision makers in case the patients were not able to sign); 3) read the medical and nurse charts (including systematic 4 to 8 hours RASS assessments whatever the patient's status: sedated or not sedated, intubated or not intubated) to check for the presence of any item before the research assessments, during the 24 previous hours.

Standardized neuropsychological assessment of delirium, adapted for critically ill patients (Reference standard)

A standardized method for diagnosing delirium was used, based on the *French translation (1) of the American Psychiatric Association's fifth edition of the Diagnostic and Statistical Manual of Mental Disorders (DSM-5).* The complete method has been detailed elsewhere (2). Briefly, attention, awareness, working memory and executive functions were tested by the MOntreal Cognitive Assessment (MOCA) (3). Long term memory was assessed by the Dubois' 5 words test (4). Orientation in space and time was tested by simple questions. Finally, the patients' expressive and reception language abilities were tested by the LAnguage Screening Test (LAST), a tool validated for the assessment of language in patients with stroke in the emergency setting which were considered as conditions close to critical illness and an ICU setting (5). These tests were performed in intubated patients using supportive materials with large printed words, letters and numbers (see below).

1. American Psychiatric Association. DSM-5 manuel diagnostique et statistique des troubles mentaux, vol. 5; 2015.
2. Eloi A, Perrigault F: Contribution à l'évaluation rapide des fonctions cognitives dans le cadre du diagnostic de confusion mentale en réanimation. Montpellier, France: Université de Montpellier; 2016.
3. Nasreddine ZS, Phillips NA, Bedirian V, et al.: The Montreal Cognitive Assessment, MoCA: a brief screening tool for mild cognitive impairment. J Am Geriatr Soc 2005; 53:695-699
4. Dubois B, Touchon J, Portet F, et al.: "The 5 words": a simple and sensitive test for the diagnosis of Alzheimer's disease. Presse Med 2002; 31:1696-1699
5. Flamand-Roze C, Falissard B, Roze E, et al.: Validation of a new language screening tool for patients with acute stroke: the Language Screening Test (LAST). Stroke 2011; 42:1224-1229

University of Montpellier

Department of Speech and Language Therapy

2, rue de l'école de Médecine

34000 Montpellier, France

Standardized neuropsychological assessment of delirium,

adapted for critically ill patients

PATIENT ASSESSMENT FORM n°.... ; date:........

LAST NAME: **First name:** **Date of birth:** **Gender:**
Observations:
Time:

<table>
<tr><td>Orientation</td><td colspan="2">Where are we? …... Which season is it?...…....................</td></tr>
<tr><td>Dubois' words</td><td colspan="2">Learning (Designation of the word for each semantic category): Lion:... Apricot:... Shirt:... Face:... Red:...
Recall 1 (if failed, repeat): Lion:... Apricot:... Shirt:... Face:... Red:...</td></tr>
<tr><td>LAST</td><td>Production
• denomination
◦ Phone:....
◦ pineapple:....
◦ pen:....
◦ crocodile:...
◦ fork:...
• repeat
◦ mathematics:............. (cat:……...)
◦ the postman brings a letter to the neighbor:……......
(hello:……...)
• automatic series: count from 1 to 10: …………..</td><td>Understanding:
• designation :
◦ rabbit:...
◦ spoon:...
◦ cigarette:...
◦ eye:...
• executive orders:
◦ show the ceiling :...
◦ do not take the glass but the pen:..
◦ put one hand on the head, then a finger on the nose:...</td></tr>
<tr><td>Recall 2 (Dubois)</td><td colspan="2">Lion:... Apricot:... Shirt:.... Face:.... Red:..</td></tr>
<tr><td>MOCA</td><td colspan="2">Right side span: 2 1 8 5 4 :…..... Back side span: 7 4 2 :……..
Letters : FBACMNAAJKLBAFAKDEAAAJAMOFAAB
.. …. .. .. …... .. …</td></tr>
<tr><td></td><td colspan="2">Delirious: yes no</td></tr>
</table>

Helpers for intubated “non verbal”patients

Orientation to place, where are you?

At the hotel

At the hospital

At home

Orientation to season, which season is it?

Spring

Winter

Autumn

Summer

Dubois 5 words: immediate recognition

tiger – bean – vest – face – blue

lion – apricot – shirt – face – red

lion – peach – pants – mouth – yellow

Adaptation: LAST production denomination 1

palm

pineapple

banana

Adaptation: LAST production denomination 2

pencil

blind

pen

Adaptation: LAST production denomination 3

phone

intercom

television

Adaptation: LAST production denomination 4

snake

crocodile

croquette

Adaptation: LAST production denomination 5

spoon

wheelbarrow

fork

Adaptation: LAST Repeat

A – B – C – D – E – F – G

– H – I – J – K – L – M –

N – O – P – Q – R – S – T

– U – V – W – X – Y – Z

Adaptation: LAST automatic series production

5 - 8 - 3 - 9 - 6 - 4 - 7- 0 - 2 -10 -1

Dubois words: delayed recognition

cat – red – apple – pull-over

– hand – lion – apricot –

shirt – tiger – bean –

face – leg – yellow
